# Low birth weight and its associated factors in East Gojjam Zone, Amhara, Ethiopia

**DOI:** 10.1186/s40795-022-00621-9

**Published:** 2022-10-31

**Authors:** Birhanie Muluken Walle, Adeyemi O. Adekunle, Ayodele O. Arowojolu, Tesfaye Tolessa Dugul, Akiloge Lake Mebiratie

**Affiliations:** 1 Department of Obstetrics and Gynecology, College of Medicine, Pan African University Life and Earth Sciences Institutes, University of Ibadan, Ibadan, Nigeria; 2 Department of Medical Physiology, College of Medicine and Health Sciences, Addis Ababa University, Addis Ababa, Ethiopia; 3 Department of Obstetrics and Gynecology, College of Medicine, University College Hospital, University of Ibadan, Ibadan, Nigeria; 4 Department of Obstetrics and Gynecology, College of Health Sciences, School of Medicine, Debre Markos University, Debre Markos, Ethiopia

**Keywords:** Low birth weight, Dietary diversity scores, Food consumption score, Multiple micronutrients, Dietary diversity

## Abstract

**Introduction:**

Low birth weight is a global public health problem, with 15–20% of all births globally, described by weight at birth of less than 2500 g ensuing fetal and neonatal mortality and morbidity, poor cognitive growth, and an increased risk of chronic diseases later in life. The prevalence is critical in East Africa where about 11% have low birth weight out of 54% of neonates whose weight was measured at birth. There are many causes of low birth weight, including early induction of labor or cesarean birth, multiple pregnancies, infections, diabetes, and high blood pressure. Moreover, socioeconomic factors and unhealthy dietary habits could contribute to low birth weight in areas with poor intake of a diversified diet. This study has indicated the association between poor dietary diversity and low birth weight in the study area for the first time.

**Methods:**

An institutional-based cross-sectional study was conducted on eligible 423 pregnant women recruited from Gestational Age of less than 17 weeks until delivery where the birth outcomes were recorded in health institutions in randomly selected five Woredas in East Gojjam Zone, Amhara, Ethiopia from June 2019 to December 2020. Questionnaires were used to collect data on socio-economic-demographic, dietary diversity scores, and food consumption scores.

**Results:**

The study found a prevalence of low birth weight of 9.6%, low dietary diversity score of 53.2%, low food consumption score of 19.7%, and preterm delivery of 9.1%. Ever attended school and a higher level of education (diploma and above) decreased the risk of low birth weight with an Adjusted Odds Ratio (AOR) of 0.149 (0.024, 0.973) *P* ≤ 0.042; 0.059 (0.007, 0.513) *P* ≤ 0.007; whereas low dietary diversity score group and low food consumption group increased the risk of low birth weight with AOR 2.425 (1.342, 6.192) *P* ≤ 0.011and 2.983 (1.956, 9.084) *P* ≤ 0.044 respectively.

**Conclusion and recommendation:**

Participants with no formal education, no diploma, and above (no college or university training/degree), low diversity score group, and low food consumption group had an increased risk of low birth weight. Therefore the use of a diversified diet, educating women to a higher educational level, and health education on the intake of a diversified food rich in multiple micronutrients are recommended as strategies that will ameliorate the occurrence of low birth weight.

**Supplementary Information:**

The online version contains supplementary material available at 10.1186/s40795-022-00621-9.

## Introduction

Low Birth Weight (LBW) is defined as weight at birth less than 2500 g. It is a major public health problem, with a prevalence of 15 to 20% worldwide. Various studies were conducted to estimate the prevalence and determinants of LBW [1]. The prevalence of LBW in various studies conducted in Ethiopia ranged from 6 to 29% [2]. The consequences of LBW include fetal and neonatal mortality and morbidity, poor cognitive development, and an increased risk of chronic diseases later in life. Moreover, current studies have found that LBW also increases the risk for non-communicable diseases such as diabetes and cardiovascular disease later in life [1]. LBW was also associated with female children, caesarean section, first child order, and preterm birth [3]. Furthermore, the sex of the infant, pregnancy-induced hypertension, ANC follow-up, prematurity, parity, and residence were found to be associated with LBW. However, most of these studies found undecided results, especially on the dietary factors associated with LBW. In addition, maternal nutrition is a modifiable risk factor of public health importance that can be integrated into efforts to prevent adverse birth outcomes, particularly among low-income populations. Therefore, understanding the relationship between maternal nutrition and birth outcomes may provide a basis for developing nutritional interventions that will improve birth outcomes and long-term quality of life reducing mortality, morbidity, and healthcare costs [4]. Moreover, dietary therapy for pregnant mothers' specific diet and nutrition might supply to condense the risk of LBW. Enhanced education also enabled women to prefer diets and career engagements that might also warrant them to decide on healthy dietary choices. Socio-demographic-economic factors including maternal age, educational status, occupation, residence, family income, and sex of the newborn are also associated with adverse birth outcomes [5, 6].

A low dietary diversity score was associated with higher odds of infant LBW whereas healthy dietary patterns (consumption of fruits and vegetables in addition to the conventional macronutrients) during pregnancy were associated with lower odds of infant LBW. Studies suggest that higher dietary diversity, nutritional status before and during pregnancy, and maternal anthropometric characteristics may be protective of LBW in addition to the socio-economic characteristics. According to the World Health Organization, poor maternal nutritional status during pregnancy confers greater risk for LBW in developing countries [7]. Whereas, adequate food or nutrient intake offers maternal nutrient reserves that can serve as nutritional stores during pregnancy. Even though it is functional to segregate the impact of specific nutrients or foods, the outcomes might be inadequate to describe the multifaceted behavior of food consumption and nutrient interactions during pregnancy [8]. Therefore, dietary diversity and dietary patterns rather than a single nutrient or food group might be useful to comprehend the association between overall maternal nutrition adequacy during pregnancy and birth outcomes principally in areas where diet assessments are complex and frequently complex [9]. Studies indicated that consumption of a diverse diet from different food groups and sources during pregnancy is a vital approach to developing dietary quality and micronutrient status during pregnancy [10].

Dietary diversity is recognized as a measure of diet quality (consumed variety of food from different food groups per day or week), nutrient adequacy, and MicroNutrient (MN) utilization with a prominent effect on adverse birth outcomes like LBW, Small for Gestational Age (SGA) and Pre Term Delivery (PTD) [11–13]. Though it is important to evaluate individual MN, it is challenging due to the complex behavior of food consumption and nutrient interactions as micronutrients are consumed naturally as a combination of nutrients and fibers [14]. Therefore, grouped Dietary Diversity Scores (DDS) and Food Consumption Scores (FCS) than single Micronutrient (MN) studies are useful for determining the overall maternal MN status and their effect on adverse birth outcomes. Moreover, single MN studies are too resource-demanding [7] to apply in Low and Middle-Income Countries (LMICs) where frequent MN deficiency has resulted in LBW, SGA, and PTD [6, 7]. This deficiency occurs in women, with no access to MN-rich fruits, vegetables, and animal products, [15] who are exclusively surviving on grains and tuber-based diets with high energy and protein but low essential amino acid and MN content [16]. Therefore, to ensure optimal nutritional status, the inclusion of diversified foods in the diet is preferred before even food fortification and MN supplementation [12]. A study indicated that the realization of more than four dietary diversity scores during pregnancy was inversely associated with the risk of maternal anemia, LBW, and pre-term birth. On the other hand, women with lower DDS during pregnancy had higher odds of delivering a higher proportion of infants with LBW compared to those who had medium and higher DDS [17]. Socio-economic-demographic factors have been also reported to affect LBW in addition to poor dietary diversity [11]. This study evaluated primarily low dietary diversity and its association with LBW using DDS and FCS which are proxy indicators for MN deficiency as described by World Food Program (WFP) and Food and Agricultural Organization (FAO) [2, 10]. Previously, information obtained from Food Frequency Questionnaires (FFQs) was also used to assess DDS, FCS, and their association with adverse birth outcomes [4, 12]. Earlier studies indicated the prevalence of low DDS in the study area as 55% [18] with an overall MMN deficiency of 20 to 48% [19]. However, poor DDS and FCS association with LBW has not yet been conducted in the study area and FCS was conducted for the first time. Hence, this study can be used also as a baseline study in the study area on the association between DDS, FCS and LBW on pregnant women.

### Objective of the study

This study determined the prevalence of low birth weight and its associated factors, including low dietary diversity and food consumption score, in East Gojjam Zone, Amhara, Ethiopia.

## Methods

A facility-based cross-sectional study was conducted between June 2019 to December 2020 on eligible consenting 423 pregnant women of GA < 17 weeks in health institutions from five randomly selected Woredas, namely Debre Markos, Dejen, Machakel, Hulet Ej Enese, and Awabel that are present in East Gojjam Zone, Amhara Regional State, Ethiopia. The sample size was determined using single population proportion formula assuming the prevalence of overall MN deficiency or poor DDS in the study area to be either 48 or 55% respectively [18]. Therefore it was calculated to be *n* = (Z_1-α/2_)^2^P(1-P)/d^2^ = 1.96*1.96*0.48*0.52/0.0025 = 383; with the addition of a 10% non-response rate, the total sample size was 423. The sample was collected based on the proportion of pregnant women in the randomly selected Woredas and continued follow-up until birth. The data were collected using a structured questionnaire about participants’ socio-economic-demographic factors, a daily and weekly dietary intake questionnaire [2, 13, 14], and translated into the Amharic language. The tool for collecting data was pretested with 5% of the respondents that were not included in the study and trained midwives working in the antenatal clinic of the corresponding health institutions have collected the data.

**Fig. 1:**
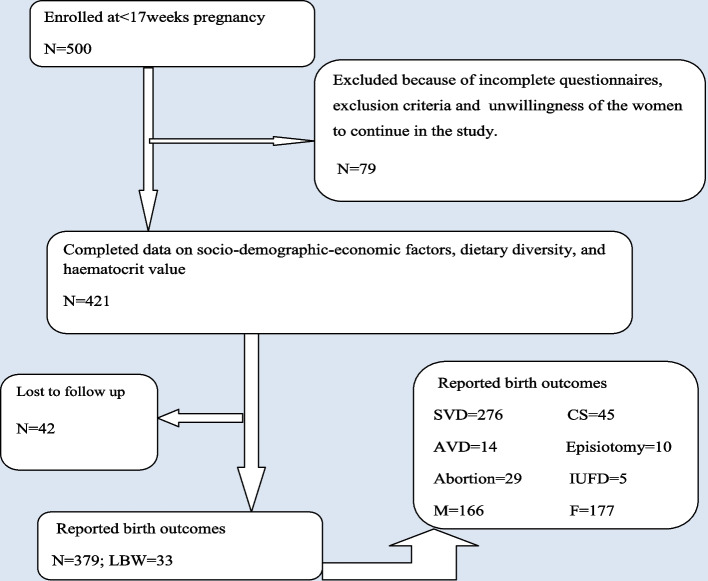
Framework on the participants' progress from < 17 weeks GA up to delivery. (SVD and AVD represent Spontaneous and Assisted Vaginal Delivery while CS, IUFD, M, and F signify Caesarian section, Intra Uterine Fetal Death, Male and Female, Low birth weight = 33 which is 9.6% in proportion)

To determine factors associated with LBW two indexes were developed from the food frequency questionnaires, the Dietary Diversity Score (DDS) and Food Consumption Score (FCS) which were categorized into the Dietary Diversity Score group (DDSg) and Food Consumption Group (FCG) as low and adequate or high. These were done from the ten food groups (mentioned below in Table 2) that were recommended for women of reproductive age including pregnant women. DDS was calculated as the sum of the number of food groups consumed by each participant during the previous 24 h with its value ranging between 1 up to 10 and a low DDSg < 5 while adequate or high ≥ 5 DDS value. The pregnant women were interviewed to respond to all the foods that they had eaten during the previous 24 h. Moreover, FCS was determined by adding the product of the weekly food group frequency and the weight of each food group. The weight was estimated based on the nutritional density of the food groups for fat, energy, protein, and MN content and bioavailability (weight of 0.5 for sugary snacks and oils; 1 for vegetables and fruits;2 for grains, 3 for pulses, 4 for meat, chicken, egg, dairy products, and fish). Moreover, FCG was categorized as low FCG ≤ 35; and adequate or high FCG ≥ 35.5. The cutoff points were developed by WFP and adopted based on the study area context (assuming weekly consumption of pulses and grains only as low FCG = 7*2 (for grains) + 7*3(for pulses) = 35; as grains and cereals are the common food groups consumed in the poor dietary diversity setup) [2, 10]. The entire progress framework of the study from the base to the end line birth outcome record was illustrated in Fig. 1. EpiData was used to enter the data and exported to IBM SPSS version 20 for analysis.

### Variables, inclusion, and exclusion criteria

LBW is regarded as an outcome variable while individual socio-economic-demographic, DDS, and FCS factors were independent variables. In addition, all consenting pregnant women (GA < 17 weeks starting from day one of their Last Menstrual Period [LMP]) who presented for ANC and planned to deliver in the selected hospitals or health centers within the study period were included. Whereas, all women with multiple pregnancies, chronic hypertension, or previous history of pre-eclampsia, severe anemia, chronic renal, liver, and gastrointestinal diseases that are likely to affect MN status, HIV/AIDS, diabetes mellitus, and poor obstetric histories were excluded [20, 21].

### Operational definition

**Low Birth Weight (LBW)** is defined as a birth weight of less than 2500 g regardless of gestational age [22].

**The dietary Diversity Score (DDS)** represents the number of different food groups consumed by each pregnant woman within 24 h. It is a qualitative measurement of food consumption, used as a proxy indicator of micronutrient adequacy at an individual level [18]. It is a continuous variable with values ranging from one to ten and pregnant women consuming at least five of the food groups per day have the minimum required diversified diet [12].

**The dietary diversity score group (DDSg)** is a categorical variable derived from the DDS where a DDSg value of less than five is considered inadequate or low and a value of more or equal to five is considered adequate or high DDSg [12, 23].

**Food Consumption Score (FCS)** another index developed by the World Food Programme [23]and asks about the weighted frequency of consumption over a week for cereals and tubers, pulses, vegetables, fruit, meat and fish, milk, sugar, and oil multiplied by the weight of each food groups out of the ten recommended food groups by FAO and WFP [24]. The weight is proportional to the nutritional density i.e. the content of micronutrients, energy, essential fat, and amino acids in each food group. Animal products have the highest weight (4) while sugary snacks and oils have the lowest (0.5). Those pregnant women with the highest consumption of food from animal, vegetable, and fruit origins will have an adequate or high FCS of more than 35.5 [23].

**Food consumption group (FCG)** is a categorical variable derived from FCS where FCG of less than35 is considered as low while a value of more or equal to 35.5% is considered as adequate or high FCG [23].

**Multiple Micronutrients (MMN)** are drug supplements that contain three or more trace elements or vitamins and minerals in a single preparation [25].

### Data analysis

The collected data were coded and entered into Epi-Data cleaned, exported to IBM SPSS, and used for data analysis. Frequency distribution for categorical variables, and mean with standard deviation for continuous variables were determined. A bivariate followed by multivariable logistic regression was used to identify the association between DDS, FCS, and SED factors with LBW at a *P* < 0.05 level of significance. Model fitness was checked using Hosmer and Lemeshow statistic test at p > 0.05.

## Results

A total of 421 study participants were included in the baseline study making a response rate of 99.5%. The prevalence of LBW and PTD were 9.6 and 9.1% respectively. The average age of the participants was 26.3 ± 4.8 years, age at first pregnancy was 22.5 ± 3.8 years, Mid Upper Arm Circumference (MUAC) 24.5 ± 2.2 cm, Body Mass Index (BMI) 21.73 ± 3.0 kg/m^2^. More than three fourth (83.1%) of study participants lived in urban areas. The vast majority of the pregnant women were Amhara in ethnicity (99%), married (96%), Orthodox Christian in religion (95.5%), 20–29 years of age (73.3%), and with no health insurance (75.6%). Most of the participants (85.5%) attended school from primary to a higher level. Moreover, a larger proportion of the participants can read and write (87.1%), with more than a quarter of the participants (26.1%) holding a Diploma and above. The male-headed household was 38.4% while the female-headed 4.6%. Among the study participants, 47.3% of pregnant women were housewives. The majority (96.6%) of the participants were below the age of 30 years and pregnant for the first time, while 19.4% were adolescents pregnant and 12.5% had low BMI values. Moreover, the low DDSg has a prevalence of 53.2% while low FCG of 19.7% as depicted in Tables 1 and 2.

**Table 1:** Socio-Economic-Demographic (SED) and other factors in East Gojjam Zone, Amhara, Ethiopia, 2020

SED factors		Categorized variables	Frequency	%	Cum. %
Place of residence		Rural	71	16.9	16.9
		Urban	350	83.1	100.0
Marital status		Married	403	96.0	96.0
		Single	17	4.0	100.0
Ever attended school?		Yes	360	85.5	85.5
Highest level of education completed		Cannot read and write	54	12.9	12.9
		Can read and write	24	5.7	18.7
		Primary education 1–5	36	8.6	27.3
		Junior secondary 6–8	46	11.0	38.3
		Senior secondary 9–12	113	27.0	65.3
		TVET	36	8.6	73.9
		Diploma and above	109	26.1	100.0
Head of the (House Hold) HH		Male-headed	158	38.4	38.4
		Female-headed	19	4.6	43.1
		^a^Equal decision-making role	234	56.9	100.0
Ethnicity		Amhara	417	99.0	99.0
		Oromo	4	1.0	100.0
Age at the first pregnancy		13–19	80	19.4	19.4
		19.9–24	205	49.8	69.2
		25–29	113	27.4	96.6
		≥30	14	3.4	100.0
Occupation		Housewife and job seeker	193	57	57
		Farmer and alcohol seller	47	14	71
		Civil servant	98	29.0	100
Having health insurance		Yes	100	24.4	24.4
Religion		Orthodox	402	95.5	95.5
		Muslim	16	3.8	99.3
		Protestant	3	.7	100.0
BMI Group		15–18.4	44	12.5	12.5
		18.5–25	263	74.9	87.5
		> 25.1	44	12.5	100.0
MUAC in cm		≤21	27	6.6	6.6
		≥22	381	93.4	100.0
Mean age of the participants		26.3 ± 4.8 years			
Birth outcomes	LBW	9.6%			
	PTD	9.1%			

^a^Equal decision-making role is to mean that both males and females in a household have equal decision-making influence

**Table 2:** Dietary diversity and food consumption scores in East Gojjam Zone, Amhara, Ethiopia, 2020

Ser. no	Food items consumed per week	Frequency	%	Weight	Days	FCS
**1**	Pulses (beans, peas, or lentils)	403	95.7	3	7	21
**2**	Special diet (fasting food, vegetarian)	11	2.6	1	2	2
**3**	Egg	187	44.4	4	2	8
**4**	Meat or chicken	81	19.2	4	2	8
**5**	Vegetables	287	68.2	1	3	3
**6**	Fruits	208	49.4	1	2	2
**7**	Fish	20	4.8	4	1	4
**8**	Dairies ( milk, cheese, and yogurt)	117	27.8	4	7	28
**9**	Grains, bread, pasta, rice, white roots	380	90.3	2	7	14
**10**	Snacks, cakes, pastries, or sugary drinks	197	46.8	0.5	3	1.5
Index	Categorized index	Frequency	%	Remark		
DDS	Low DDSg	224	53.2	Prevalence of low DDS		
	Adequate or high DDSg	197	46.8			
FCS	Poor FCG (≤ 35)	83	19.7	Prevalence of low FCS		
	Adequate or high FCG (≥ 35.5)	338	80.3			

Regarding dietary intake, low DDSg 53.2 and low FCG of 19.7% were recorded where pulses 95.7%, and whole grains 90.3%, were the most commonly consumed food items while fish 4.8%, and special dietary requirements 2.6%, are the least consumed as indicated in Table 2 below.

At the end line of data collection, a total of 379 (89.6%) participants' birth outcomes were recorded as indicated in Fig. 1. Male neonates account for 48.4%, females 51.6%, Spontaneous Vaginal Delivery (SVD), 80%, CS 13%, LBW 9.6%, PTD 9.1% and 11% previous pregnancy failure were recorded, with about half of the participants attended at least four ANC visits.

### Logistic regression results

The multiple logistic regression analysis was conducted between LBW and DDS, FCS, or other associated factors as indicated in Table 3 below, low FCG and low DDSg increased while ever attending school and a higher level of education (diploma and above) decreased the risk of LBW with AOR of = 2.98 (1.96, 9.08) *P* ≤ 0.04); 2.43 (1.34, 6.19) *P* ≤ 0.01; 0.15 (0.02, 0.97), *P* ≤ 0.04and 0.06 (0.01, 0.51), *P* ≤ 0.01 respectively. There was also an insignificant increase in the risk of LBW due to regular coffee consumption.

**Table 3:** Logistic regression between low birth weight and predictor variables in East Gojjam Zone, Amhara, Ethiopia, 2020

SN	Predictor variable	sig	Exp(B)COR (CI 95%)	AOR 95% CI (*P*-value)	H&LT
1	FCS	0.001	.959 (0.942, 0.977)	#	0.129
2	Low FCG	0.001	7.812 (3.89, 15.691)	2.983 (0.001)*	1
3	Moderate FCG (FCG = 35.5)	0.001	3.13 (1.563, 6.267)	#	1
4	DDS	0.001	0.624 (0.522, 0.746)	#	0.499
5	Low DDSg	0.001	3.805 (2.063, 7.018)	2.425 (0.011)*	0.23
6	Ever attend school	0.019	0.504 (0.024, 0.937)	0.149 (0.024,0.937),(0.042)*	0.872
7	Higher education	0.002	(0.295) (0.014, 0.974)	0.059 (0.007, 0.513),(0.007)*	0.59
8	Education on MMN	0.027	0.432 (0.205, 0.91)	#	0.11
9	Coffee use	0.018	1.691 (1.096, 2.608)	#	0.729

Predicted Probability is of Membership for LBW (≤ 2499); Reference is the last categorical response of the predictor variable. # = the *P*-value was ≥ 0.05 and not significant. *H & LT* Hosmer and Lemeshow goodness-of-fit test

## Discussions

In this study, the proportion of LBW, low DDSg, and 'poor' FCG have been identified to be 9.6%, 53.2%, and 19.7% respectively. This indicated more than half of the participants have a DDS of less than 5 and 19.7% have an FCS less than or equal to 35. Moreover, almost half of the participants have an age group between 19.9 to 24 years with no health insurance in a quarter of them and low BMI and MUAC prevalence of 12.5 and 6.6% respectively. Previous studies conducted in Ethiopia indicated a LBW between 6% to 29.1% [2] and this study indicated also an LBW of 9.6%. Most of the participants in the study were urban residents, married, below grade 12 level of education, male than female-headed, and housewives.

In this study, it was observed that an increase in FCS resulted in a decrease of LBW by 4% and there was a significant increase of LBW in participants with a low FCG. Moreover, lower DDSg and 'poor' FCG were associated with a high risk of LBW owing to low MN and food insecurity as shown by these variables. Another study in Tanzania also indicated higher DDSg and FCG association with lower risk of SGA, PTB, and LBW [26]. A study indicated that those who attended formal education or higher level of education reduced risks of low DDSg and FCG [27] indicating their indirect role on the weight of the newborn. In addition, low DDSg during pregnancy was also associated with higher odds while dietary patterns were considered as healthy and traditional during pregnancy with lower odds of LBW in a study conducted in Ghana [7]. In this study, regular coffee consumption also increased the LBW by 69.1% (*P* ≤ 0.05) in the bivariate analysis. Another study also reported that maternal caffeine consumption association with major negative pregnancy outcomes including LBW [28]. Though macronutrient deficiency could also result in the risk of LBW, this was not observed in this study as 90–95% of the participants were consuming energy-dense grains and pulses. This further explains the association of LBW with MN deficiency due to low consumption of diet of animal, fruit, and vegetable origin.

Women in the developing world are more likely to have staple diets due to limited variation in available foodstuffs, putting them at the risk of MN deficiencies. Population groups that are most affected by MN deficiencies are those that subsist on refined cereal grain or tuber-based diets. Such diets provide energy and protein but often lack some critical MN, and essential amino acids [13, 21]. Low DDSg indicated a proportional deficiency of MMN ranging from five to eleven including riboflavin, niacin, folate, vitamin B_12_, calcium, and iron as indicated in previous studies [18, 23] which could explain the LBW associated with this low DDS and FCS. There was 19.5% low FCG and 53.2% low dietary intake (out of the ten food groups 53.2% consume less than five food items per day indicating inadequate intake of diversified food resulting in MN deficiency). Similar findings of DDS were reported in previous studies in the zone indicating also deficiency of a diversified food consumption score in more than half of the participants [18]. In addition, for the first time, this study evaluated FCS in the study area and obtained a low FCG of 19.5% (FCG ≤ 35) with the pulses and grains taken in higher proportions while meals like fish, meat, and chicken were taken in lower proportions. Previous studies in the Mota town had also reported a similar 18.5% low FCG [29] and MN deficiencies are common in populations that survive on refined cereal grain or tuber-based diets having high energy but low essential amino acids content [16]. Similarly in this study pulses and whole grains were the most commonly consumed food items by a majority of the participants, thus predisposing them to MN malnutrition and increased risk of LBW. Previously, a mean DDS of 3.68 (± 2.10) with a comparable low DDSg prevalence of 55% has been recorded in the study area where the commonly consumed dietary groups were legumes, nuts, and seeds (85.5%) followed by starchy staples (64.7%) [18]. In addition, a different study also indicated that cereals were the most commonly (96%) consumed food group while fish, eggs, and fruits were the least consumed food group in Ethiopia [30]. In a study from Nigeria, in all of the states, more than 10% of households had also poor or borderline FCG [31]. Previous studies also indicated that women having low DDS or less consumption (1%) of Eggs and meat had 80% greater chances of a LBW resulting in adverse birth outcomes with long-term consequences in a child [32]. Another systematic review also indicated the association of LBW with poor dietary diversity [33].

## Conclusion

In this study, low DDSg, and low FCG (which are proxy indicators of MN deficiency), frequent coffee consumption, and absence of health education on MMN increased the risk of LBW. However, those participants who ever attended school and a higher level of education reduced the risk of LBW.

### Recommendation

This study recommends health education about the intake of diversified nutrients from animals, vegetables, and fruits than only grains and pulses as this will help pregnant women to have a sufficient amount of multiple MN from these diets which could reduce the risk of LBW. This has to be done to promote the prenatal, antenatal, and postnatal consumption of the required MNs for healthy birth outcomes. Besides educating the women and encouraging them to attain higher levels of education will also reduce the risk of low DDSg and 'poor' FCG that result in LBW. The household member has to be told also the role of a diversified rather than a repetitive food schedule during pregnancy as there is no single food that can contain all the nutrients.

### Limitations of the study

Though the sample size of the study was sufficient enough to draw those conclusions stated above, the study is limited by the less number of participants from rural areas. Moreover, in the majority of study health centers, the investigator couldn't get ultrasound results due to a lack of equipment. This resulted in a sole determination of the gestational age of the patients’ by the recall of the first day of the last menstrual period without confirmatory tests from ultrasound scanning. The COVID-19 virus pandemic also resulted in a loss of follow-up of participants in the end-line data collection of the birth outcomes. Due to resource limitations, the study has failed to measure the nutrient intake from the foods consumed.

## Supplementary Information

**Additional file 1.**

**Additional file 2.**

## Data Availability

The datasets used and or analyzed during the current study are available from the corresponding author on sensible request.
