# Supplementary material for: Low birth weight and its associated factors in East Gojjam Zone, Amhara, Ethiopia
Source: BMC Nutr. 2022 Oct 31;8:124. doi: 10.1186/s40795-022-00621-9 (PMC9620599; doi:10.1186/s40795-022-00621-9)
Supplement: Supplementary file 2 — Additional file 2. [file 40795_2022_621_MOESM2_ESM.docx]

# . Annex I: Questionnaire English and Amharic version

1.1. MICRONUTRIENTS AND THEIR ASSOCIATION WITH PREGNANCY OUTCOMES IN EAST GOJJAM ZONE, AMAHARA, ETHIOPIA.

Introduction/respondent’s consent

Dear respondent,

We are conducting a study on the association between micronutrient deficiency and pregnancy adverse outcomes. We would appreciate your assistance in filling this questionnaire and freely expressing your honest opinion. Your responses would be made nameless such that it can’t be traced or linked to you. Thank you for the collaboration.

1. Section A-Socio economic and demographic factors of the mother.

Name of data collector____________________ Code of data collector_________

| SN | Question |  |
| --- | --- | --- |
|  | Woreda | 1. Name __________________ 2. Code \| \| \| |
|  | Hospital | 1. Name__________________ 2. Code\| \| \| |
|  | Women's code | 1. Name_____________________ 2. Code \| \| \| \| |
|  | ID no. of women | \| \| \| \| \| \| \| \| |
|  | Telephone No. | ________________________ |
|  | Place of residence | 1. Rural 2. Urban |
|  | Resident Kebele Name | 1. Name_____________ 2. Code\| \| \| |
|  | In what month and year were you born? | 1. Month\| \| \| 2. Year\| \| \| \| \| |
|  | Age of women (as at last birthday-in yrs) | \| \| \| |
|  | Marital status | 1. Married/living together 2. Single/never married/never lived together 3. Divorced/separated 4. Widowed |
|  | Have you ever attended school? | 1. Yes 2. No |
|  | Highest level of education completed | 1. Do not read and write 2. Read and write 3. primary 4. junior secondary 5. Senior secondary 6. Tertiary |
|  | Economic status  How many of the following animals does this household own? | 1. Cows/ Bulls \| ­­­ \|___\| 2. Other Cattle\| ­­­ \|___\| 3. Horses/ Donkeys/ Mules \| ­­­ \|___\| 4. Goats\| ­­­ \|___\| 5. Sheep\| ­­­ \|___\| 6. Chicken/ Poultry\| ­­­ \|___\| 7. Beehives \| \| |
|  | Does any member of this household own any agricultural land? | 1. Yes 2. No |
|  | How many hectares of agricultural land do members of this household own? | 1. \| ­­­ \|___\| 2. Don't know |
|  | Does your house hold have   1. Electricity 2. Radio 3. Television 4. A non-mobile telephone 5. A computer 6. A refrigerator 7. A table 8. A chair 9. A bed with a mattress 10. A lamp | 1. 1=Yes; 2=No 2. 1=Yes; 2=No 3. 1=Yes; 2=No 4. 1=Yes; 2=No 5. 1=Yes; 2=No 6. 1=Yes; 2=No 7. 1=Yes; 2=No 8. 1=Yes; 2=No 9. 1=Yes; 2=No 10. 1=Yes; 2=No |
|  | Does this house hold own   1. A watch 2. A mobile phone 3. A bicycle 4. A motorcycle 5. An animal drawn cart 6. A car or truck? 7. A boat with a motor 8. A Bajaj | 1. 1=Yes; 2=No 2. 1=Yes; 2=No 3. 1=Yes; 2=No 4. 1=Yes; 2=No 5. 1=Yes; 2=No 6. 1=Yes; 2=No 7. 1=Yes; 2=No 8. 1=Yes; 2=No |
|  | Does any member of this household have a bank account? | 1=Yes  2=No |
|  | Estimated family monthly income per month in birr | \| ­­­ \|___\| ­­­ _\|___\| |
|  | Head of the house hold | 1. Husband 2. Wife 3. Son or daughter 4. Husband and wife   Others please specify_________ |
|  | Who will decide on buying and selling of houses, land cattle? | 1. Husband 2. Wife 3. Son or daughter 4. Husband and wife   Others please specify_________ |
|  | Religion | 1. Orthodox 2. Islam 3. protestant 4. Others please specify_________ |
|  | Ethnicity | 1. Amhara 2. Oromo 3. Tigre 4. Others, please specify_________ |
|  | Do you have any sons or daughters to whom you have given birth who are alive but do or do not live with you? | 1. Yes 2. No |
|  | 1. How many children are alive but do not live with you? 2. How many children are alive and live with you? | 1. Children elsewhere\| \| \| 2. Children at home\| \| \| 3. Total \| \| \| |
|  | Have you ever given birth to a boy or girl who was born alive but later died? | 1. Yes 2. No |
|  | How many children have died? | \| \| \| |
|  | What was the result of previous pregnancy? | 1. Live 2. Deceased 3. My first pregnancy |
|  | Do you know how many ANC follow ups you will have? | 1. Yes 2. No |
|  | How many ANC follow ups you will have? | 1. <4 2. 4 to 7 3. >7 |
|  | How many live children do you have so far? | \| \| |
|  | How long have you stayed before the first pregnancy? | \| \| |
|  | Total number of pregnancies? | \| \| |
|  | Total number of deliveries? | \| \| |
|  | How long between this and last pregnancy in months | \| \| |
|  | Your age during the first pregnancy in yrs | \| \| |
|  | How many months pregnant are you? | \| \| \| |
|  | When you got pregnant, did you want to get pregnant at that time? | 1=Yes  2=No |
|  | Did you want to have a baby later on or did you not want any more children? | 1=Later  2=No more/ None |
|  | Have you ever had a pregnancy that miscarried, was aborted, or ended in a stillbirth? | 1=Yes  2=No |
|  | When did the last such pregnancy end? | Month\| \| \|  Year\| \| \| \| \| |
|  | When did your last menstrual period start? | 1=\| \| \|days  2=\| \| \| Weeks |
|  | Did you see anyone for antenatal care for this pregnancy? | 1. Yes 2. No |
|  | Whom did you see? | 1. Doctor 2. Nurse 3. Midwife 4. Health officer 5. Health extension worker 6. Traditional birth attendant   Others(specify)___________ |
|  | How many weeks pregnant were you when you first received antenatal care for this pregnancy? | 1. \| \| \| Weeks 2. Don't know |
|  | How many times do you plan or told to receive antenatal care during this pregnancy? | 1. \| \| \| times 2. Don't know |
|  | During (any of) your antenatal care visit(s), were you told about the signs of pregnancy complications or danger sign of pregnancy? | 1=Yes  2=No |
|  | Which signs of pregnancy complications were you told about? | 1. Vaginal bleeding 2. Vaginal gush of fluid 3. Severe head ache 4. Blurred vision 5. Fever 6. Abdominal pain 7. Convulsion   Others(specify)______________ |
|  | During any of your anenatal visit were you told about birth preparedness plan? | 1. Place of birth 2. Supplies needed for birth 3. Emergency transportation 4. Money/ Emergency fund 5. People to support during afterbirth 6. Potential blood donors   Others_________________ |
|  | As part of your antenatal care during this pregnancy, were any of the following done at least once?   1. Was your blood pressure measured? 2. Did you give a urine sample? 3. Did you give a blood sample? 4. Did any health worker give you Nutritional Counseling? | 1. 1=Yes; 2=No 2. 1=Yes; 2=No 3. 1=Yes; 2=No 4. 1=Yes; 2=No |
|  | During this pregnancy, were you given or did you buy any iron tablets? | 1= Yes  2=No |
|  | During the whole pregnancy, for how many days did you take the tablets? | 1.Days \| \| \| \|  2. Don't know |
|  | During this pregnancy, did you take any  drug for intestinal worms? | 1= Yes  2=No  3=Don't know |
|  | 1. Do you think you are treated equally in the hospital? | 1. Yes 2. No |
|  | Weight before pregnancy (kg) |  |
|  | Occupation (Employment status) | 1. House maid 2. Farmer 3. Alcohol producer('Tela' or 'Arekie') 4. Civil servant 5. unemployed 6. Others please specify_________ |
|  | Had any new cravings for particular foods or beverages? | 1=Yes  0=No |
|  | Had any new aversions for particular foods or beverages? | 1=Yes  0=No |
|  | What is the main source of water for drinking, cooking and hand washing for members of your household? | 1=Piped water  2=Dug water  3=Water from spring  4=Rain Water  5=River water  Others(Specify)_________________ |
|  | Where is that water source located? | 1=In own dwelling  2=In own yard/plot  3=Else where |
|  | How long does it take to go there, get water, and come back? | \| \| \| \|Minutes |
|  | Who usually goes to this source to fetch the water for your household? | 1=Adult woman  2=Adult man  3=Female child(<15yrs)  4=Male child (15yrs)  Others (specify)______________ |
|  | In the past two weeks, was the water from this source not available for at least one full day? | 1=yes  2=No  3=Don't know |
|  | Do you do anything to the water to make it safer to drink? | 1=yes  2=No  3=Don't know |
|  | What do you usually do to make the water safer to drink? | 1=Boil  2=Add chlorine/water guard  3=Use water filter  4=Let it stand and settle  Others(specify)_________________ |
|  | What kind of toilet facility do members of your household usually use? | 1=Flush or Pour flush toilet  2=Pit latrine  3=Composting toilet  4=No facility/Bush/Field  Others(specify)_________________ |
|  | Do you share this toilet facility with other households? | 1=Yes  2=No |
|  | Including your own household, how many households use this toilet facility? | 1≤10  2≥10  3=Don't know |
|  | Where is this toilet facility located? | 1=In own dwelling  2=In own yard/plot  3=Else where |
|  | What type of fuel does your household mainly use for cooking? | 1=Electricity  2=Kerosine  3=Charcoal  4=wood  5=Animal dung  Others(Specify)____________ |
|  | Is the cooking usually done in the house, in a separate building, or outdoors? | 1=House  2=A separate building  3=Outdoors  Others(Specify) |
|  | Do you have a separate room which is used as a kitchen? | 1=Yes  2=No |

1. Section B- Maternal, fetal and neonatal anthropometry.

|  | Length of gestation or gestational age (in Weeks) | \| \| \| |
| --- | --- | --- |
|  | Weight of the women in kg\| \| \| height in cm\| \| \| \| |  |
|  | BP Systolic\| \| \| \|mmHg Diastolic\| \| \| \| mmHg. |  |
|  | Women mid upper arm circumference(cm) | \| \| \| |
|  | Inter pregnancy intervals (months). | \| \| \| |
|  | Ultrasound scan result fetal sex |  |
|  | Ultrasound scan result (last menstrual cycle) fetal age (in weeks). | \| \| \| |
|  | Ultrasound scan result fetal weight gm) for gestational age(1=normal or better; 0= below normal) | \| \| \| |
|  | Ultrasound scan result fetal head circumference cm) for gestational age (1=normal or better; 0= below normal) | \| \| \| |
|  | Ultrasound scan result fetal length (in cm) for gestational age (1=normal or better; 0= below normal) | \| \| \| |

1. Section C-Environmental and cultural factors.

|  | Climate zone of the residence of the women | 1. Tropical(kolla) 2. Woina dega (Subtropical zone) 3. Dega (Cool zone) |
| --- | --- | --- |
|  | Residence Kebele's name. | Name_____________ |
|  | Can you mention parasitic infestations you exposed during pregnancy | 1. Malaria Yes No 2. Intestinal parasites Yes No 3. specify if Others__________ |
|  | Were you taking any alcoholic drinks during pregnancy? | 1. Almost everyday 2. Frequently(>once/week) 3. Less frequently(<once a week) 4. Never drink |
|  | Were you smoking during this pregnancy? | 1. Yes(Frequently) 2. Less Frequently 3. Never |
|  | Were you chewing khat during this pregnancy? | 1. Yes(Frequently) 2. Less Frequently 3. Never |
|  | Was there a family member who smokes at home? | 1. Yes(Frequently) 2. Less Frequently 3. Never |
|  | Is there any food taboo during pregnancy? | 1. Yes 2. No |
|  | Do you expose yourself to sunlight during pregnancy? | 1. Yes(Frequently) 2. Less Frequently 3. Never |

1. Section-D Questions used to assess behavioral determinants (belief, self-efficacy and social norms).

|  | Please tell us if you agree with the following statements (1=agree, 0=disagree). | |
| --- | --- | --- |
|  | My consuming right types and amount of food during pregnancy is extremely important for my health and my unborn child. | 0=disagree  1=agree |
|  | My consuming right types and amount of food during pregnancy is extremely important for my unborn child’s brain/education and ability to earn | 0=disagree  1=agree |
|  | I can manage to follow the recommendations of 5 varieties of food to be consumed during pregnancy | 0=disagree  1=agree |
|  | I can manage to follow the recommendations of adequate amounts of food to be consumed during pregnancy | 0=disagree  1=agree |
|  | My family members and community people will be angry if I consume the right types and amounts of food during pregnancy^2^ | 0=disagree  1=agree |
|  | I cannot consume the recommended types and amounts of food as we are poor people | 0=disagree  1=agree |
|  | It is too costly to obtain the recommended types and amounts of foods for my consumption during pregnancy^2^ | 0=disagree  1=agree |
|  | It is a good use of our family’s money to ensure the right types and amounts of foods during pregnancy. | 0=disagree  1=agree |
|  | It is a good use of our family’s money to ensure the right types and amounts of foods as contributes to the future welfare of the child and family | 0=disagree  1=agree |
|  | In my family and community, I am expected to consume so many varieties and such large amount during pregnancy | 0=disagree  1=agree |
|  | (Behavioral determinants were assessed based on mothers agree or disagree to the questions. Each item was given a score of 1 (agree) or 0 (disagree). Range score: 0-9 ;Questions with reverse coded.) |  |

1. Section-E. Questions used to assess husbands’ support

|  | Please tell us if you agree with the following statements (1=agree, 0=disagree) | |
| --- | --- | --- |
|  | My husband does not purchase diversified nutritious foods and does not ensure that I have these foods available^2^ | 0=disagree  1=agree |
|  | My husband reminds and encourages me to consume the recommended quantity of diversified foods daily | 0=disagree  1=agree |
|  | My husband helps me to ensure that there are enough tablets of IFA and Calcium at home | 0=disagree  1=agree |
|  | My husband reminds me to take one tablet of IFA and on tablet of Calcium daily | 0=disagree  1=agree |
|  | My husband does not remind /helps me to take rest for 2 hours during the day in addition to sleeping at night^2^ | 0=disagree  1=agree |
|  | My husband and family members make me lifting heavy work load during pregnancy | 0=disagree  1=agree |
|  | My husband reviews my weight gain chart and helps me find ways to gain enough weight during pregnancy | 0=disagree  1=agree |
|  | My husband calls the health worker on mobile if I have any difficulties to do any of the above. | 0=disagree  1=agree |
|  |  |  |

1. Section-F. Questions used to assess determinants of micronutrient level

| 1. 1 | Do you have any special dietary requirements (eg. vegetarian, vegan, allergies)? If yes, please list them: | ____________ |
| --- | --- | --- |
| 1. 2 | 2.1. Quality of diet |  |
|  | 1. Do you eat meat or chicken regularly? Number of days __________________ 2. Do you eat pulses regularly? Number of days ___________________ 3. Do you eat vegetables regularly? Number of days ___________________ 4. Do you eat Fruits regularly? Number of days ___________________ 5. Do you eat fish regularly? Number of days ___________________ 6. Do you eat special fasting diet regularly? Number of days ___________________ 7. Do you eat dairy products regularly? Number of days ___________________ 8. Do you eat egg regularly? Number of days ___________________ 9. Do you eat whole grains regularly? Number of days ___________________ 10. Do you eat snacks regularly? Number of days ___________________ | 1=Yes 0=No  1=Yes 0=No  1=Yes 0=No  1=Yes 0=No  1=Yes 0=No  1=Yes 0=No  1=Yes 0=No  1=Yes 0=No  1=Yes 0=No  1=Yes 0=No |
| 1. 3 | What is your |  |
|  | 3.1.Did/do you take folate/folic acid supplements in pre-pregnancy and in early pregnancy (first 12weeks)? | 1=Yes 0=No |
|  | 3.2. Do you get regular exposure to the sun (face, arms and hands for at least 10-15 mins per day)? | 1=Yes 0=No |
|  | 3.3. Has the doctor/nurse tested your hemoglobin (level of iron in the blood)? | 1=Yes 0=No |
|  | 3.4. Have you taken a Zinc supplement |  |
|  | 3.4. *(Health care professional to complete)* If yes, is it more than 110 g/l?  Enter the value: ................. | 1=Yes 0=No |
| 1. 4 | Have you heard of micronutrients before? | 1=Yes 0=No |
| 1. 5 | Do you have health education about minerals and vitamins before? | 1=Yes 0=No |
| 1. 6 | Have you ever had Vaccinations for vitamin A, D, Vitamin B12 or Iron before? | 1=Yes 0=No |

1. Checklist for the laboratory evaluation

| SN | Question |  |
| --- | --- | --- |
|  | Woreda | 1. Name __________________ 2. Code \| \| \| |
|  | Hospital | 1. Name__________________ 2. Code\| \| \| |
|  | Women's code | 1. Name_____________________   2. Code \| \| \| \| |
|  | ID no. of women | \| \| \| \| \| \| \| \| |
|  | Telephone No. | ________________________ |
|  | Place of residence | 1. Rural 2. Urban |
|  | Micronutrient or other parameters | Amount |
|  | Haemoglobin conc. (gm/dl) |  |
|  | Haematocrit value(HCT%) |  |

1. Section-G. Questions used to assess pregnancy outcomes

|  | Pregnancy outcome | Duration/ amount/ length |
| --- | --- | --- |
|  | Day of delivery | \| \| \|Day  \| \| \|Month  \| \| \| \| \|Year |
|  | Gestational age at birth(weeks) | \| \| \| |
|  | Birth Weight(BW in gm) | \| \| \| \| \| |
|  | Head circumference in cm | \| \| \| |
|  | Baby length in cm | \| \| \| |
|  | Apgar score | \| \| \| |
|  | Duration of pregnancy | \| \| \|weeks |
|  | Duration of labor | \| \| \|hrs |
|  | PTD(Preterm Delivery) | 1=Yes 2=NO |
|  | Stillbirth | 1=Yes 2= NO |
|  | First-week neonatal mortality | 1=Yes 2=NO |
|  | Maternal mortality | 1=Yes 2= NO |
|  | Baby sex | 1=M 2= F |
|  | Which danger signs of newborn health were observed? | 1=Feeding less  2=Too hot or too cold  3=Too sleepy  4=Convulsion  5=Fast breathing  6=Umblical red /PU  7=Pus in eye  8=Fever  Others(specify)___________ |
|  | Mode of delivery | 1=Vaginal  2=caesarean section  Other(specify)_____________ |
|  | Need for augmentation of delivery | 1=Yes  2=No |
|  | Postpartum hemorrhage |  |
|  | Other adverse outcomes if any |  |
|  | The total number of ANC follow-ups done? |  |
|  | Health professional/s at the ANC who see the client? |  |
|  | Who assisted with the delivery? |  |
|  | How long after delivery the did woman stay in the hospital? | 1=\| \| \| hrs  2=\| \| \| days  3=\| \| \|weeks  Don't know |
|  | Type of vaccination given like TT | 1=Yes  2=No |
|  | During this pregnancy, was there treatment for intestinal worms? | 1=Yes  2=No |
|  | Any danger signs of maternal health occurred? | 1=Heavy vaginal bleeding  2=Fever  3=Smelly vaginal bleeding  4=Depression  Others(Specify)________ |
|  | Next date of appointment to the hospital | \| \| \|Day  \| \| \|Month  \| \| \| \| \|Yrs |

1. Any comment or suggestions by the women about the study_________________________________

Thank you for your time!!
